# The Downstream Impacts of High Drug Costs for PrEP Have Hindered the Promise of HIV Prevention

**DOI:** 10.1017/jme.2022.35

**Published:** 2022

**Authors:** Kenyon Farrow

**Keywords:** PrEP, HIV, Drug Pricing, Disparities, Access

## Abstract

Prior to the recent introduction of generic TDF/FTC in the U.S., access to pre-exposure prophylaxis (PrEP) for HIV was greatly limited due to the downstream effects of the high cost of the medication. This article argues that despite drug copay cards and patient assistance programs, the promise of drastically reduced HIV diagnoses has never been fully realized, and more policy reforms on drug pricing are needed to make ending the HIV epidemic a reality.

When tenofovir disoproxil fumarate/emtricitabine (TDF/FTC) was approved for the use of prevention of HIV in 2012 by the U.S. Food and Drug Administration, many saw it as a watershed moment that could move us toward truly ending the HIV epidemic in the U.S. and globally. But 10 years later, that has hardly happened. In the United States, the wealthiest nation in the world, the rollout of PrEP has been nothing short of an abysmal failure.

Black people make up approximately 13% of the total U.S population, and about 44% of HIV diagnoses annually.^1^

And despite this disparity, we are also among the smallest population of people who are prescribed PrEP. According to a 2019 CDC analysis, only 11.2% of all PrEP users in the U.S. were Black.^2^ And while more than 50% of HIV diagnoses occur in the Southern states (which is still where most Black people in America live), most of the Southern states (except for Louisiana and Arkansas) have not expanded Medicaid under the Affordable Care Act. A 2020 study found that states that had expanded Medicaid *and* had a PrEP Access Program in the state had a 99% higher PrEP use than states that had neither, leaving many of the Southern states with large Black populations and large HIV prevalence rates with low access to PrEP and corresponding low usage.^3^

While many people place the blame of low PrEP use on the lack of knowledge, interest or initiative of the consumer, far less work has been done to assess the impact of the list price of the medication on the ability of users to access PrEP as well as stay on PrEP. Debates about drug pricing often come down to the amount a patient has to pay out of pocket when they fill a prescription. But high drug list prices for antiretroviral medications, including those used for prevention, still impact access to these drugs downstream, even when the patient has low or no out-of-pocket costs. And while patient assistance programs can help cover some of those out-of-pocket costs for people who are not insured or cannot afford the prescription co-pays, they have not been enough to increase equitable access. The high list prices of the initial brand name drugs for PrEP have dictated PrEP delivery systems in the U.S., including how PrEP was marketed, clinical guidelines to determine PrEP eligibility, and prior authorization requirements from payers.

The list price of PrEP medication and its impact on the ability to scale up use to have public health benefit was described in research literature as early as 2008, four years before the drug was approved. A study published that year assessed the cost effectiveness of implementing a PrEP program targeting men who have sex with men in New York City. The model to determine cost-effectiveness used the 2007 wholesale list price of TDF/FTC, which the authors note was $31USD per pill, making the monthly supply $930USD.^4^ A 2011 analysis from *AIDS* found that the $13,416 wholesale list price of Truvada would undermine the cost effectiveness of PrEP implementation in the United States.^5^ And in 2012, Philpott noted the “use of antiretroviral drugs for HIV prevention can be an effective public health intervention if and only if (1) the drugs are made widely available to those at highest risk; (2) the drugs are used consistently; (3) the drugs are provided as part of a comprehensive counseling program, including frequent follow-up testing; and (4) widespread risk compensation (e.g., decreased condom use or increased numbers of sexual partners) does not occur.”^6^ These four criteria for PrEP scale up have all been threatened because of the high list price of the medications.

On May 16, 2019, Gilead Sciences CEO Daniel O’ Day testified in front of Congress on the pharmaceutical company’s reasoning for keeping the price of TDF/FTC at approximately $1800 for a 30-day supply. O’Day offered that despite the high cost of the medication, it did not impede access, stating, “We offer a wide range of programs to help ensure that people have access to Truvada when they need it. For example, 98% of people who use our copay Assistance Program have no out-of-pocket costs. In fact, according to the CDC’s own estimates when taking our programs into account, less than 1% of Americans who would benefit from PrEP are in need of financial assistance to obtain Truvada.^7^ Furthermore, O’Day contradicts his own testimony about the impact of the price of Truvada on patients when his written testimony also stated a major barrier to PrEP access is “Insurance benefit design that places a significant cost-sharing burden on patients.”

In other words, according to O’Day, the price set by Gilead Sciences for PrEP is not the problem. The problem is the insurers’ cost mitigation efforts, like higher copays and out of pocket expenses for PrEP users. But the issue that causes the policies of insurance companies still boils down to cost, and it is the public that pays in the end.

The idea that the public, who invested heavily in the development of TDF/FTC for both treatment and prevention through studies funded by the National Institutes of Health, is made whole through Gilead’s patient assistance programs, is unfounded. In order to be eligible, a patient must have a prescription, proof of income, proof of residency, be uninsured, and make no more than 500% of the federal poverty level (approx. $70k). If a patient applies through Gilead’s Advancing Access website, conditional approval for 30 days is usually instant (sometimes there’s a delay or denial if income or insurance status recently changed). If denied, a patient can fax a paper application (4 pages with supporting documents such as a month’s worth of pay stubs or insurance termination letter), in which approval is usually 48 hours or less. If a PrEP patient has Advancing Access but does not renew within 12 months of the designated expiration date, the patient is not eligible to apply online and, instead, must submit a paper application.

In essence, poor and working class PrEP users who depend on Gilead’s program are made to jump through more administrative hoops to access PrEP, which may play a role in the abysmal use rates. Also, PrEP users are at the mercy of their providers or PrEP navigators to either alert them to these options and help them fill out the paperwork for approval. If a patient is not made aware of this program, then they may just not pick up the prescription.

In addition to manufacturer assistance programs being a woefully insufficient and inefficient way to ensure access, very little attention has been paid to the ways in which the high list price of Gilead’s two brand name drugs approved for PrEP impacts payer policy, public health social marketing and education programs, and even clinical guidelines.

Until the list price of generic TDF/FTC fell in early 2021, PrEP users on most private insurance plans were still required to go through prior authorization every month to get the prescription, which created administrative burden both on the provider and the patient. Often, prior authorization is used by insurers when there is more than one drug option, as a way to ensure that providers are giving a clinical reason for a patient needing a more expensive drug when a cheaper, equally effective option is available. And yet, for many years when TDF/FTC was the only option, patients were still forced to go through variable and arbitrary prior authorization process, with clinicians being required to document HIV risk or other clinical criteria for PrEP use. Many patients were also required to use mail order specialty pharmacies to access PrEP, which may not be the most convenient option for people living in places where they don’t want a medication to be mailed to their home for privacy reasons (e.g., students in campus housing or living at home with parents, etc.). Ultimately the high list price of the drug incentivized insurers to more tightly and narrowly restrict access as a cost containment mechanism.

When PrEP was first being implemented, much of the focus of public health programs in major U.S. cities was to create social marketing and community education programs focused almost exclusively on promoting PrEP to gay and bisexual men. While gay and bisexual men, and other men who have sex with men, make up the majority of new diagnoses in the U.S., they are not the only people who are vulnerable to HIV infection. Cisgender women account for about 20% of HIV diagnoses in the U.S. but only make up 7% of PrEP users.^8^ To be sure, public health programs also prioritized targeted marketing and education programs for men who have sex with men due to the vastly higher prevalence rates among this population. But one question that should be explored by historians, bioethicists, and other social scientists is whether the decision to nearly exclusively market PrEP to gay and bisexual men (until there was pushback from cisgender women and transgender activists) was at least in part due to the cost of the medication itself. Or were the very complicated sexual risk assessments in the early clinical guidelines that made it so that many Black and Brown men and women were missed as not having enough risk factors was either consciously or unconsciously written knowing a broader population of PrEP users would stretch already grossly underfunded city, county and state HIV/STD public health programs.^9^

Drug pricing has become a major issue in American politics, with Americans across the political spectrum and every walk of life agreeing that Congress should do more to control for the cost of health care and all medications. And as we reach the 10th anniversary of the approval of the first drug approved for PrEP, manufacturer assistance and donation programs are not a panacea for the system that has kept the cost of the medication absurdly high.

Many studies have concluded that the disproportionately higher HIV incidence among Black communities in the U.S. does not correlate with higher incidence of sexual or drug use risk behaviors.^10^ Instead, higher incidence of HIV among racial and ethnic minorities with less access to adequate healthcare has created conditions where more people with HIV do not know their status and are not virally suppressed. Because the first clinical guidelines for PrEP eligibility were written for providers to take individual risk assessments, many Black and Brown women and men who have sex with men would not meet the bar for individual risk, while their risk for HIV acquisition based on community level viral load and other factors, greatly increased their risk.

Luckily, several structural changes recently have helped turn the tide on some of these upstream structural barriers to PrEP access. The U.S. Services and Preventative Task Force A grade rating for PrEP in 2019^11^ helped reduce or eliminate cost sharing for PrEP. In 2021 as generic TDF/FTC became more widely manufactured and reduced the price of a 30-day supply from $1800 for brand name PrEP down to $40 for a generic, many of these restrictive payer and clinical guideline policies have been removed. With generic TDF/FTC now widely available at such a low cost, pharmacies have begun allowing patients to take home a 90-day supply. More and more marketing and education campaigns are reaching cisgender women and transgender people. And the CDC in late 2021 relaxed its clinical guidelines for providers, essentially allowing PrEP to be prescribed to any sexually active adult who is HIV negative.^12^

Drug pricing has become a major issue in American politics, with Americans across the political spectrum and every walk of life agreeing that Congress should do more to control for the cost of health care and all medications. And as we reach the 10th anniversary of the approval of the first drug approved for PrEP, manufacturer assistance and donation programs are not a panacea for the system that has kept the cost of the medication absurdly high.

To suggest that the only solutions to high drug costs is to help cover the out-of-pocket cost of medications is short sighted at best, for it does nothing to address the ways in which drug price impacts the very health care system that patients access downstream — from prior authorization to whom a particular intervention is marketed to and how restrictive clinical guidelines are written. The community of PrEP users, activists, and providers must come together to push for changes in our ability to control prices for medications. Any plan to end the epidemic will fail without public policy reforms on drug pricing to adequately address the upstream the cost of PrEP medications, because the high retail price limits what public health and health care systems can do to increase utilization downstream.

## Note

The author was a moderator of a panel in 2020 for Viiv Healthcare’s annual summit for community advocates.
